# Cellular responses of BRCA1-defective and triple-negative breast cancer cells and in vitro BRCA1 interactions induced by metallo-intercalator ruthenium(II) complexes containing chloro-substituted phenylazopyridine

**DOI:** 10.1186/1471-2407-14-73

**Published:** 2014-02-07

**Authors:** Tidarat Nhukeaw, Pornvichai Temboot, Kanidtha Hansongnern, Adisorn Ratanaphan

**Affiliations:** 1 Laboratory of Pharmaceutical Biotechnology, Department of Pharmaceutical Chemistry, Faculty of Pharmaceutical Sciences, Prince of Songkla University, Hat-Yai, Songkhla 90112, Thailand; 2 Department of Chemistry, Faculty of Science, Prince of Songkla University, Hat-Yai, Songkhla 90112, Thailand

**Keywords:** Ruthenium, BRCA1, Triple-negative, Cell cycle, Apoptosis, BRCA1, Ubiquitination

## Abstract

**Background:**

Triple-negative breast cancer (TNBC) is defined by the absence of expression of estrogen receptor, progesterone receptor and human epidermal growth factor receptor 2. Breast cancers with a BRCA1 mutation are also frequently triple-negative. Currently, there is a lack of effective therapies and known specific molecular targets for this aggressive breast cancer subtype. To address this concern, we have explored the cellular responses of BRCA1-defective and triple-negative breast cancer cells, and in vitro BRCA1 interactions induced by the ruthenium(II) complexes containing the bidentate ligand, 5-chloro-2-(phenylazo)pyridine.

**Methods:**

Triple-negative MDA-MB-231, BRCA1-defective HCC1937 and BRCA1-competent MCF-7 breast cancer cell lines were treated with ruthenium(II) complexes. The cytoxoxicity of ruthenium-induced breast cancer cells was evaluated by a real time cellular analyzer (RTCA). Cellular uptake of ruthenium complexes was determined by ICP-MS. Cell cycle progression and apoptosis were assessed using propidium iodide and Annexin V flow cytometry. The *N*-terminal BRCA1 RING protein was used for conformational and functional studies using circular dichroism and in vitro ubiquitination.

**Results:**

HCC1937 cells were significantly more sensitive to the ruthenium complexes than the MDA-MB-231 and MCF-7 cells. Treatment demonstrated a higher degree of cytotoxicity than cisplatin against all three cell lines. Most ruthenium atoms were retained in the nuclear compartment, particularly in HCC1937 cells, after 24 h of incubation, and produced a significant block at the G2/M phase. An increased induction of apoptotic cells as well as an upregulation of p53 mRNA was observed in all tested breast cancer cells. It was of interest that BRCA1 mRNA and replication of BRCA1-defective cells were downregulated. Changes in the conformation and binding constants of ruthenium-BRCA1 adducts were observed, causing inactivation of the RING heterodimer BRCA1/BARD1-mediated E3 ubiquitin ligase activity.

**Conclusions:**

This study has revealed the ability of ruthenium complexes to inhibit cell proliferation, induce cell cycle progression and apoptosis. Ruthenium treatment upregulated the marker genes involved in apoptosis and cell cycle progression while it downregulated BRCA1 mRNA and replication of HCC1937 cells. Our results could provide an alternative approach to finding effective therapeutic ruthenium-based agents with promising anticancer activity, and demonstrated that the BRCA1 RING domain protein was a promising therapeutic target for breast cancers.

## Background

Triple-negative breast cancers (TNBCs) are defined by the lack of expression of an estrogen receptor (ER), a progesterone receptor (PR), and human epidermal growth factor receptor 2 (HER2). They represent approximately 15% of all breast cancers and account for a higher percentage of breast cancer related mortality detected in African and African-American women [1]. TBNCs share genetic and morphological abnormalities with basal-like breast cancer (BLBC), a subgroup of breast cancers defined by gene expression profiling [2]. Breast cancers with a BRCA1 mutation are also frequently triple-negative and basal-like. TNBC has important clinical implications including a typical high grading, and a high rate of proliferation. However, TNBC has a less favorable clinical outcome in terms of the nature and progression, compared with other subtypes of breast cancer. TNBC responds to conventional chemotherapy but relapses more frequently than hormone receptor-positive types, and exhibits poorer outcomes or prognosis [3]. Poly (ADP-ribose) polymerase (PARP), EGFR, and mTOR inhibitors are among the therapeutic agents being studied in patients with TNBC and BRCA1-associated breast cancers [4]. However, the most recent outcomes have not been fruitful. In the adjuvant setting, anthracyclines and taxanes are the drugs of choice for TNBC patients [5]. Neoadjuvant regimens, including platinum drugs in combination with taxane, can achieve high pathologically complete response (pCR) rates in TNBC [6]. Currently, an approach to the use of platinum agents, cisplatin and carboplatin to treat TNBC are being assessed in clinical trials, based on the dysfunction of BRCA1 and its pathways associated with a specific DNA-repair defect [7]. TNBC patients with decreased BRCA1 expression can be sensitized to cisplatin [8]. In other word, cisplatin treatment has improved outcomes for some TNBC patients [9].

Although it is widely used in cancer chemotherapy, application of cisplatin is somewhat limited because of its severe toxicity and also because of the development of drug resistances [10]. Moreover, cisplatin-induced secondary mutations in the tumors of BRCA1 mutation carriers have been shown to confer resistance to such a platinum-based drug [11]. These limitations have consequently motivated extensive investigations into alternative metal-based cancer therapies. Ruthenium compounds seem to be the most promising alternatives to platinum complexes for new-generation therapies [12]. Ruthenium compounds offer potential benefits to the antitumor platinum(II) complexes such as reduced toxicity, no cross-resistance and they have a different spectrum of activity. The low toxicity of ruthenium drugs is attributable to similar ligand exchange kinetics to those of the platinum(II) complexes, and different oxidation states under physiological conditions. In addition, ruthenium is capable of mimicking iron in binding to carrier proteins such as transferrin, that has been postulated to be a specific metal (mainly iron) delivery mechanism to cancer cells that require higher iron requirement. As a result, ruthenium compounds could be well suited for cancer treatment [12-16].

**Figure 1:**
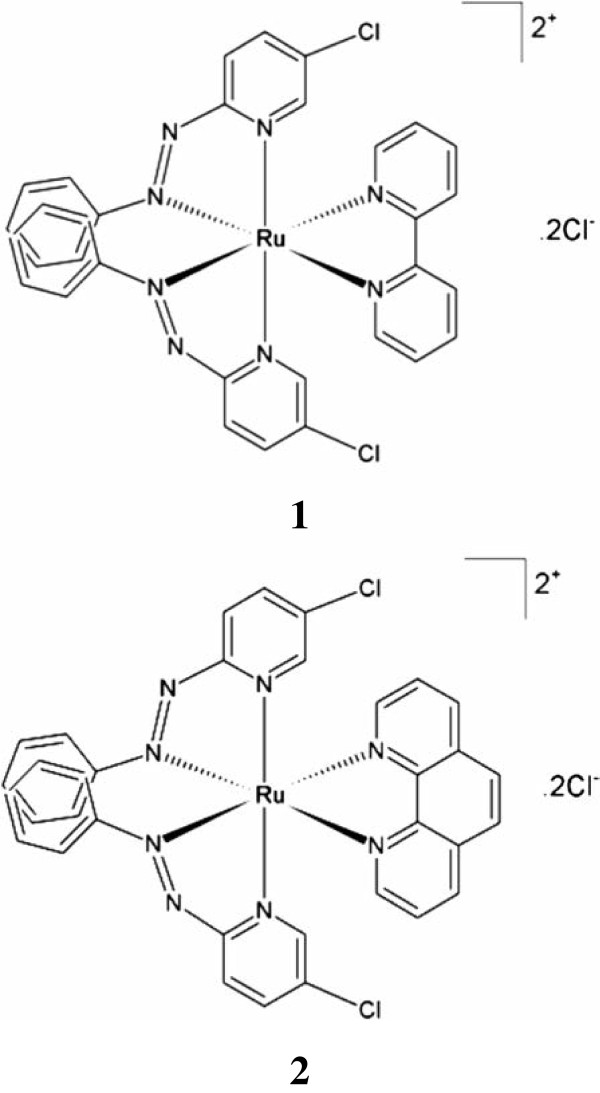
**Chemical structures of ruthenium(II) complexes with a chloro-substituted phenylazopyridine ligand.** [Ru(Clazpy)_2_bpy]Cl_2_.7H_2_O **(1)** and [Ru(Clazpy)_2_phen]Cl_2_.8H_2_O **(2)** used in this study.

Several ruthenium compounds have exhibited high cytotoxicity towards cancer cells and for inducing apoptosis [15-17]. In addition, extensive investigations of ruthenium-based compounds has mainly focused on the characterization of ruthenium-DNA adducts [12,18-20] and has paid less attention to other potential cellular targets. For ruthenium-based drug candidates, their precise mechanisms of action as anticancer activities remain comparatively unexplored. There is evidence to indicate that ruthenium compounds might directly interfere with specific proteins involved in the signal transduction pathways, cell adhesion and migration processes [21-23]. It has been demonstrated that cancerous cells with inactivated BRCA1 had a defect in the repair of DNA double strand breaks (DSBs), and this conferred hypersensitivity towards platinum-based chemotherapy drugs and radiation [24,25]. However, the effect of ruthenium compounds on the BRCA1-mediated DNA repair pathway remains unidentified, in particular, for a potential therapeutic role of the ruthenium complexes in targeting BRCA1-mediated ubiquitination in homologous recombination (HR) repair. Therefore, it is of interest to consider this metal-based compound as a therapeutic agent perhaps for aggressive triple-negative and BRCA1-defective breast cancers (TNBC). In the present study, we have explored the cellular responses to metallo-intercalator ruthenium(II) complexes with the Clazpy ligand, [Ru(Clazpy)_2_bpy]Cl_2_.7H_2_O (**1**) and [Ru(Clazpy)_2_phen]Cl_2_.8H_2_O (**2**) (Figure 1) [18], in selected BRCA1-defective and triple-negative breast cancer cells as well as by testing the possibility that the *N*-terminal BRCA1 RING domain protein was a potential biomolecular target for these ruthenium-based anticancer agents in breast cancers.

## Methods

### Cell culture

The cell lines, including human breast adenocarcinoma cells [MCF-7 (BRCA1 wild-type, ER positive); MDA-MB-231 (BRCA1 wild-type, TNBC); and HCC1937 [BRCA1 mutant (5382insC), TNBC], were purchased from the American Type Culture Collections (ATCC, Rockville, MD, USA). HCC1937 cells were grown in Roswell Park Memorial Insititute 1640 medium (RPMI 1640) (Life Technologies, Paisley, UK) without phenol red. MCF-7 and MDA-MB-231 cells were grown in Dulbecco’s modified Eagle’s medium (DMEM) (Life Technologies, Paisley, UK) without phenol red. All media were supplemented with 10% fetal bovine serum (FBS) and 1% penicillin-streptomycin. All cell lines were cultured at a constant temperature of 37°C in a 5% carbon dioxide (CO_2_) humidified atmosphere.

### Real-time cell growth profiling

The real time growth kinetics of MCF-7, MDA-MB-231 and HCC1937 cells towards the metal-complex treatments were examined using the Real-Time Cellular Analyzer (RTCA) (*x*CELLigence System, Roche Applied Science, Mannheim, Germany). RTCA utilizes an E-plate that contains interdigitated micro-electrodes integrated on its bottom. The cell number, viability, morphology and degree of adherence of cells in contact with the electrodes will affect the local ionic environment leading to an increase in the electrode impedance. This is represented as the Cell Index (CI) and reflects a calculation (via an internal system algorithm) of the frequency-dependent electrode impedance with or without attached healthy cells present on the surface of the wells. For each experiment, 100 μL of medium was added into 96-well E-plates and the background readings were recorded. A cell suspension (100 μL) at a cell density of 5 × 10^4^ cells/well was added to each well of the E-plate. The attachment, spreading and proliferation of the cells was monitored every 15 min over the following 7 h for the MCF-7 cells and HCC1937 cells and for 18 h with the MDA-MB-231 cells (this allows for cell attachment, spreading and cells entering their logarithmic growth phase). When the cells entered the logarithimic phase, the plate was removed from the RTCA machines. The cells were washed once with PBS to remove any cell debris, and either fresh medium containing various concentrations of the metal complexes or fresh medium (control) was added to each well. The plate was reinserted into the RTCA machine and the proliferation of the cells were further assessed every 15 min for the next 24 h. After a 24 h incubation time, the medium containing the metal complexes was removed, the wells were again washed once with PBS and fresh medium was added to all wells. The plate was then reinserted into the RTCA machine for a further 24 h to assess the degree of cellular recovery in the absence of the metal complexes. Experiments were performed in triplicate.

### Cellular uptake and distribution

About 5 × 10^6^ cells were seeded into 75 cm^3^ cell culture flasks. The cells were treated with **1** and **2** at their IC_50_ values (Table 1) and then incubated at 37°C in 5% CO_2_ for 2, 12, 24 and 48 h, respectively. The medium was removed and washed twice with 5 mL of PBS buffer [26]. The ruthenium content in three fractions (cytoplasm, mitochondria and nuclear fraction) was analyzed by inductively coupled plasma-mass spectrometer (ICP-MS) (Agilent Technologies, USA).

**Table 1:** **IC**_**50 **_**values (μM) for 1, 2, and cisplatin on MCF-7, MDA-MB-231 and HCC1937 cells after 24 h of treatment (data reflect the mean and ± SD of results from three separate experiments, each performed in triplicate)**

**Metal complexes**	**IC**_**50 **_**(μM)**		
	**MCF-7**	**MDA-MB-231**	**HCC1937**
Cisplatin	42.2 ± 8 ^*,**^	128.2 ± 7 ^*,**^	23.4 ± 7 ^*,**^
**1**	10.7 ± 0.6 ^*,**^	14.1 ± 0.5 ^*,**^	9.9 ± 0.2 ^*,**^
**2**	8.2 ± 0.1 ^*,**^	13.2 ± 0.3 ^*,**^	1.8 ± 0.1 ^*,**^

Statistical significance differences are indicated by *p < 0.01, compared the IC_50_ values of the same complex on cell lines, and **p < 0.01, compared the IC_50_ values of the complexes on each cell line.

### Cell cycle analysis

About 10^6^ cells were seeded into 6-well culture plates. Cells were incubated in the absence and the presence of the IC_50_ concentration of **1** and **2** for 24 h. Following incubation the cells were trypsinized, washed twice with 0.5 mL of PBS and centrifuged at 300 *g* for 5 min and then 10^6^cells were collected and fixed in cold 70% ethanol at -20°C overnight. The fixed cells were washed twice with PBS. The cell pellets were resuspended in 1 mL of PBS (100 μg/mL of RNase A, 50 μg/mL of PI, and 0.1% of Triton-X 100), and then further incubated at 37°C in the dark for 30 min. The fluorescence of 20000 cells was measured using a FACSCanto flow cytometer. The cell cycle distribution was analyzed with MultiCycle software. The proportions of cells in the sub-G1, G0/G1, S, and G2/M phases were represented as DNA histograms.

### Annexin V apoptosis detection assay

About 10^6^ cells were seeded into 6-well culture plates. Cells were incubated in the absence and the presence of the IC_50_ concentrations of **1** and **2** for 24 h. Following incubation the cells were trypsinized, washed twice with 0.5 mL of PBS and centrifuged at 300 *g* for 5 min. The pellet was resuspended in 100 mL of 1× Annexin-binding buffer. Alexa Fluor 488 Annexin V, 5 μL, and 1 μL of PI (100 μg/mL) were added to each cell suspension which were then further incubated at room temperature for 15 min. Then, 400 μL of 1× Annexin-binding buffer was added and mixed gently. Annexin V binding was analyzed on a FACSCanto flow cytometer equipped with a fluorescence emission at 530 and 575 nm using a fluorescence excitation at 488 nm.

### Cellular BRCA1 damage using QPCR

About 10^6^ cells were incubated with various concentrations of **1** or **2** at 37°C for 48 h in 5% CO_2_. Genomic DNA of the ruthenium-treated or untreated (control) cells was isolated, and the 3426-bp fragment of the BRCA1 exon 11 of the cells was then amplified by PCR, electrophoresed on 1% agarose gel, stained with ethidium bromide and then visualized under UV light [20]. The quantitative PCR (QPCR) method was used to assess the polymerase inhibiting effect of DNA ruthenation. The amplification products were quantified using a Bio-Rad Molecular Imager, and the amount of DNA amplification (%) was plotted as a function of the concentration [20].

### Real-time quantitative RT-PCR

The breast cancer cells were plated and cultured in complete medium and allowed to grow for 48 h followed by the addition of the IC_50_ concentrations of **1** and **2**. The cells were further incubated at 37°C. The cells were harvested and the total RNA was extracted from cultured cells using the RNeasy® Mini Kit (Qiagen, Germany). cDNA was obtained by reverse transcription of total RNA using QuantiTech^®^ Reverse Transcription (Qiagen, Germany). The primer sequences were as follows:

BRCA1: 5^/^-GCCAGTTGGTTGATTTCCACC-3^/^ (forward) and 5^/^-GTCAAATGTGCTCCCCAAAAGC-3^/^ (reverse)

p53: 5^/^-GGTCTCCCCAAGGCGCACTGG-3^/^ (forward) and 5^/^-AGGCTGGGGGCACAGCAGGCC-3^/^ (reverse)

p21: 5^/^-GACACCACTGGAGGGTGACT-3^/^ (forward) and 5^/^-CAGGTCCACATGGTCTTCCT-3^/^ (reverse)

β-Actin: 5^/^-GGACTTCGAGCAAGAGATGG-3^/^ (forward) and 5^/^-AGCACTGTGTTGGCGTACAG-3^/^ (reverse).

Real-time PCR reactions were then carried out in a total volume of 25 μL including 100 ng of the cDNA template, 12.5 μL of QuantiFast SYBR green PCR master mix, and the final concentration of primers of 0.5 μM. The PCR conditions were as follows: 5 min at 95°C, and 35 cycles of 10 sec at 95°C, 30 sec at 60°C. Fluorescence was measured during the annealing step on an ABI-Prism 7300 analytical thermal cycler (Applied Biosystems). Data were analyzed according to the 2^-∆∆C^_T_ method [27], and normalized by β-Actin mRNA expression in each sample. Experiments were performed in triplicate.

### Plasmid constructions, expression and purification

The *N*-terminal BRCA1 RING domain protein containing the 304 amino acid residues was prepared by PCR-mediated cloning as previously described [24]. The purified protein was identified on 12% Coomassie blue-stained SDS-PAGE and subsequently confirmed by sequencing the tryptic digested peptides.

### Circular dichroism

The *N*-terminal BRCA1 (1–304) proteins (10 μM) were prepared in deionized water, according to the Bradford assay using BSA as standard. ZnCl_2_, ruthenium polypyridyl complexes (**1** and **2**) were prepared as 1 mM stock solutions in deionized water. BRCA1 protein with and without pre-incubation of 3 mol-equivalent ratio of Zn^2+^ were treated with **1** and **2** at various concentrations. Metal-dependent folding of the protein was monitored by acquiring a CD spectrum over a range of 200–260 nm using a *Jasco J720* spectropolarimeter (Japan Spectroscopic Co., Ltd., Hachioji City, Japan). Measurements of ruthenium complex binding were carried out at 20°C using a 0.1 cm quartz cuvette. The spectrum was averaged from five separate spectra with a step size of 0.1 nm, a 2 s response time and a 1 nm bandwidth. Data were baseline-corrected by the subtraction of each metal complex concentration. The secondary structures of proteins were predicted using the CONTIN program [28]. The effect of ruthenium complex binding on the protein conformation was determined in the absence and presence of a 3 mol-equivalent ratio of Zn^2+^ to protein. The binding constant was determined as described previously [29].

### In vitro ubiquitination assay and western bloting

The ubiquitin ligase reactions (20 μL) contained 20 μM Ub, 300 nM E1, 5 μM UbcH5c, 3 μg BRCA1 or Ru-BRCA1 adduct, and 3 μg BARD1 in a buffer [50 mM Tris (pH 7.5), 0.5 mM DTT, 5 mM ATP, 2.5 mM MgCl_2_, and 5 μM ZnCl_2_]. Two separate reactions were incubated at 37°C for 3 h, and then terminated by adding an equal volume of SDS-loading dye before electrophoresis on 10% SDS-PAGE. The separated protein was then transfered to the PVDF membrane and immunodetected with anti-His_6_ HRP (Horseradish Peroxidase) conjugated (chemiluminescent method, QIAGEN) at a dilution of 1:2000 performed according to the manufacturer’s protocol. The blot was detected by chemiluminescence (SuperSignal TM, Pierce) on X-ray film. The relative E3 ligase activity of the BRCA1 adducts was quantified by normalizing the density of an apparent band of the ubiquitinated-protein conjugates to that of the parental BRCA1 as the control, using a Bio-Rad GS-700 Imaging Densitometer.

### Data processing and statistical analysis

Values are shown as the standard error of the mean unless indicated otherwise. Data were analyzed and, where appropriate, the significance of the differences between the mean values was determined using one-way ANOVA. A probability of 0.01 was deemed statistically significant. The following notation was used throughout: * *p* < 0.01, relative to control.

## Results

### Anti-proliferative effects of ruthenium(II) polypyridyl complexes with the bidentate ligand 5-chloro-2-(phenylazo)pyridine

**Figure 2:**
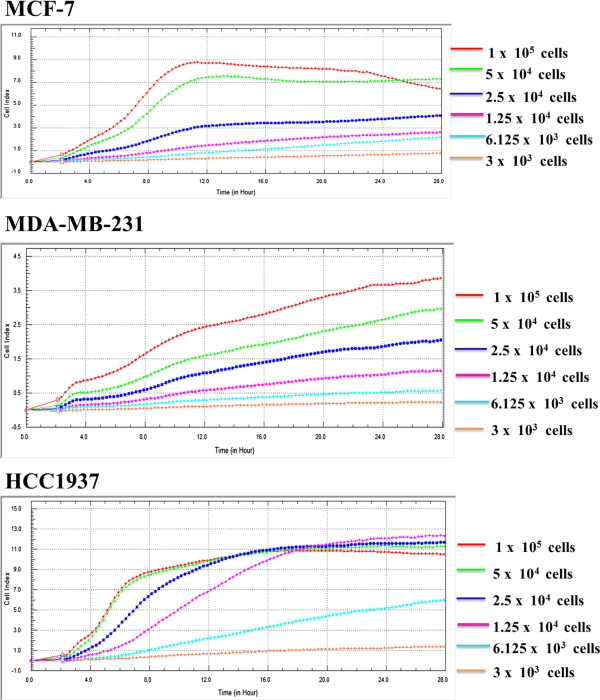
**Real time growth profiling of human breast cancer MCF-7, HCC1937 and MDA-MB-231 cells was examined using the Real-Time Cellular Analyzer (RTCA) (*xCELL*igence System, Roche Applied Science, Mannheim, Germany).** The results acquired from three separate experiments, each performed in triplicate.

**Figure 3:**
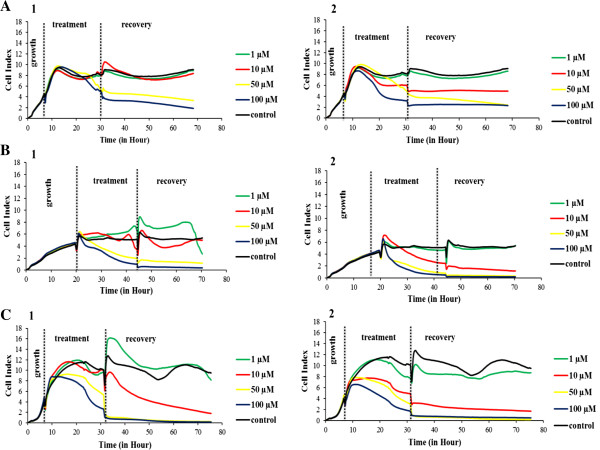
**Real-time monitoring of the ruthenium(II) polypyridyl complexes, 1 and 2, effects on human breast cancer cells using the *xCEEL*igence system.** Cells [MCF-7 **(A)**, MDA-MB-231 **(B)**, and HCC1937 **(C)**] were seeded onto the E-plate and allowed to grow prior to the introduction of the metal complexes at various concentrations. After addition of the metal complexes, cells were allowed to grow for 24 h in the presence of **1** or **2**. The complexes were then removed and fresh medium was added. Cells were then allowed to grow for 24 h to assess the recovery of cell proliferation after ruthenium treatments. The cell index (CI) was recorded every 15 min. Each concentration was performed in triplicate.

The real-time monitoring of these breast cancer cell proliferation on the 96-well E-plates were monitored at 15 min intervals from the time of plating until the cells entered their logarithmic growth phase (Figure 2), following which the cells were treated with different concentrations of the metal complexes. After treatment, the cell index (CI) values were read at 15 min intervals for 24 h. With the MCF-7 cells, it was observed that there was a rapid decrease in the CI value that occurred 5 h after treatment with 100 μM, 50 μM, and 10 μM of **2**, and a rapid decrease in the CI value at 15 h after treatment with 100 μM and 50 μM of **1** (Figure 3A). This indicated that MCF-7 cells were sensitive to both of these complexes but the rate of the responses was different. In the HCC1937 cells, there was a rapid decrease in the CI value that occurred as early as a few hours after treatment with 100 μM, 50 μM, and 10 μM of **2** and **1**, respectively (Figure 3C). However, both complexes showed a more rapidly decrease in the CI value of HCC1937 than for the MCF-7 cells. These results were supported by the MTT assay that both complexes had lower IC_50_ values in HCC1937 than MCF-7 cells (Additional file 1: Table S1). This indicated that both complexes appear to be more active against the HCC1937 cell lines than the MCF-7 cells. Furthermore, the MDA-MB-231 cells that had been treated previously with both complexes showed a rapid decrease in the CI value, that occurred as early as a few hours after treatment with 100 μM, 50 μM, and 10 μM. However, the MDA-MB-231 cells exhibited more sensitivity to **2** than **1** (Figure 3B). **2** appeared to be more active against MCF-7 and MDA-MB-231cells than **1**, indicating a higher degree of effectiveness to HCC1937 cells than the MCF-7 or MDA-MB-231 cells. Moreover, the transient increase of the CI value in all cell lines that was observed in all experiments, indicated a change in cell interactions in response to treatment before induction of cell death. The IC_50_ values at 24 h post-treatment with **1** and **2** for MCF-7, MDA-MB-231, and HCC1937 cells are summarized in Table 1. To determine whether the metal complexes have sustained effects on these cells, following their removal, the cellular recovery was assessed within a 24 h period post replacement of the metal complex medium with fresh media. The cell lines tested did not recover from the suppressive effects of both **1** and **2** at the concentration of 100 μM, 50 μM, and 10 μM, but a transient recovery was observed at lower concentrations of these complexes. However, after a few hours of recovery, the cells did die. MCF-7, HCC1937 and MDA-MB-231 cells still died when the metal complexes were removed after 24 h. This indicated that irreparable cell damage had occurred.

### Differential cellular accumulation

**Figure 4:**
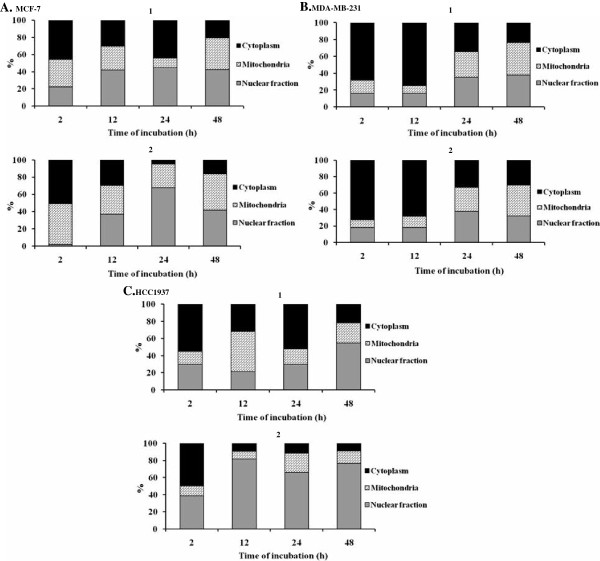
**Cellular uptake of 1 and 2 in MCF-7 (A), MDA-MB-231 (B) and HCC1937 (C) cells.** MCF-7, MDA-MB-231 and HCC1937 cells were exposed to an appropriate IC_50_ concentration of **1** or **2** (Table 1) for 2, 12, 24 and 48 h. The intracellular ruthenium content was subsequently determined by ICP-MS. Percentage localizing in the cell fragments calculated on the basis that their sum was 100%.

The distribution of ruthenium in cells after exposure to **1** and **2** is summarized in Figure 4. It was apparent that both ruthenium complexes reached the nuclear fraction of the tested breast cancer cells within 24 h of incubation. It was of interest that, in the HCC1937 cells, **2** entered and was retained in the nuclear fraction as the largest portion after 12–48 h of incubation, while **1** deposited the largest amount of ruthenium atoms in the nuclear fraction at 48 h of incubation. Our data indicated that HCC1937 cells have a preferential uptake of **2** rather than **1**. The retention of ruthenium atoms in the nuclear compartment could damage DNA, and ultimately lead to cancer cell death or apoptosis.

### [Ru(Clazpy)_2_phen]Cl_2_.8H_2_O (2) produced a significant block at the G2/M phase with prominent induction of apoptosis in triple-negative MDA-MB-231 cells

**Figure 5:**
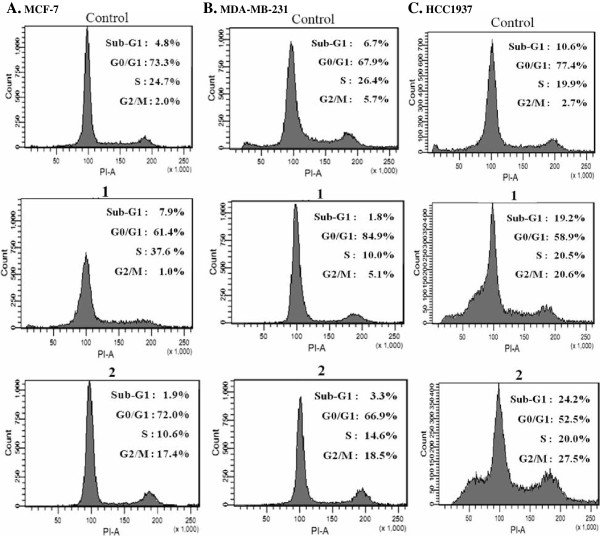
**Flow cytometric analysis of the cell cycle phase distribution in response to 1 and 2.** MCF-7 **(A)**, MDA-MB-231 **(B)**, and HCC1937 **(C)** cells were treated with appropriate IC_50_ concentrations of **1** and **2** for 24 h, and the DNA content was then analyzed by propidium iodide (PI) staining. The results were expressed as a histogram display of their DNA content (x-axis: PI fluorescence) versus counts (y-axis). The phases of the cell cycle from the left to the right were sub-G1-phase, G0/G1-phase, S-phase, and G2/M-phase, respectively.

The effects of the ruthenium complexes at their IC_50_ on cell cycle progression were analyzed by propidium iodide flow cytometry at 24 h. It was of interest, that treatment with **2**, induced G2/M cell cycle arrest, as evidenced by accumulation of cells in the G2/M of all three tested cancer cells. In particular in the HCC1937 cells, a 10-fold increase in the population of G2/M cells was observed, and there was a concomitant increase in the population of sub-G1 cells (2-fold). There was no significant alteration in the S phase observed for the BRCA1-associated breast cancer cells. However, **1** slightly diminished the number of MDA-MB-231 and MCF-7 cells at G2/M (Figure 5). These results can be interpreted to mean that **1** and **2** have distinct modulations on the arrest of cell cycle progression.

**Figure 6:**
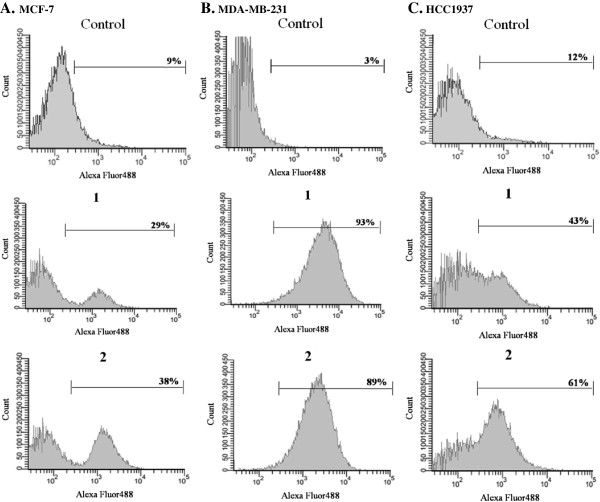
**Ruthenium(II) polypyridyl complexes induced apoptosis in MCF-7 (A), MDA-MB-231 (B) and HCC1937 (C) cells treated with appropriate IC**_**50 **_**concentrations of 1 or 2 (Table**1**) for 24 h.** Flow cytometric profiles of Annexin-V FITC staining in a representative experiment are shown.

**Figure 7:**
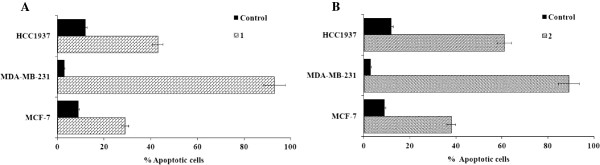
**Ruthenium(II) polypyridyl complexes induced apoptosis in MCF-7, MDA-MB-231 and HCC1937 cells treated with appropriate IC**_**50 **_**concentrations of 1 (A) or 2 (B) (Table 1) for 24 h.** The percentage of apoptotic cells was detected by analyzing for the Annexin V-FITC and PI binding using flow cytometry.

Ruthenium-induced apoptosis was assessed in all three tested cancer cells. A prominent induction of apoptosis was apparent at their IC_50_ concentrations. A significant increase in apoptotic cells in triple-negative MDA-MB-231 cells was observed with slightly less apoptotic cells in HCC1937 and MCF-7 cells, respectively (Figures 6 and 7).

### Ruthenium (II) complexes slightly blocked BRCA1 amplification

**Figure 8:**
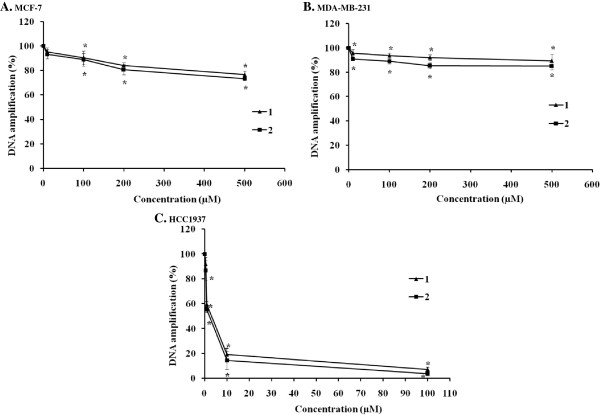
**DNA amplification of the 3,426-bp fragment of BRCA1 exon 11 after cellular treatment with the ruthenium(II) polypyridyl complexes.** MCF-7 **(A)**, MDA-MB-231 **(B)**, and HCC1937 **(C)** cells were treated with various concentrations of **1** and **2** at 37°C for 24 h before genomic DNA was isolated. Genomic DNA was then amplified with forward/reverse primers for the 3,426-bp fragment of the BRCA1 gene in a PCR reaction mixture for 30 cycles. PCR products were electrophoresed on 1% agarose gel. The gel was stained with ethidium bromide and visualized under UV illumination. Amplification products were quantified as described in the materials and methods section. The amount of DNA amplification (%) was plotted as a function of the drug concentration. The mean ± the standard error of experiments realized in duplicate is plotted. Statistical significance differences from the untreated control are indicated by * *p* < 0.01.

DNA is a key cellular target for cancer chemotherapy including platinum-based chemotherapy. Platinum drugs exert their antitumor effects by binding to DNA, thereby changing the replication of DNA and inhibiting the growth of the tumor cells. For ruthenium-based drugs, there has been some evidence demonstrating an interaction between ruthenium complexes and DNAs [18,20,30]. Our previous study has demonstrated that **1** and **2** bound to the specified DNA fragment of the human breast cancer suppressor gene 1 (BRCA1) through the intercalative mode into the base pairs of DNA, and the DNA-binding constants (K_b_) for **1** and **2** were 7.09 × 10^4^ M^-1^ and 5.19 × 10^5^ M^-1^, respectively. This data indicated that the binding affinity of these two complexes to DNA was dependent on the aromatic planarity and hydrophobicity of the intercalative polypyridyl ligand [18]. We further investigated whether the ruthenium(II) complexes with the bidentate ligand 5-chloro-2-(phenylazo)pyridine were capable of blocking BRCA1 amplification. To address this question, the QPCR method was used to monitor the progress of the DNA polymerization. Both **1** and **2** caused a similar reduction of BRCA1 amplification as the concentration of the ruthenium complexes increased (Figure 8). This implied that both **1** and **2** can blocked the replication of the BRCA1 gene as the dose increased. It was a surprise that at equimolar concentrations, this class of ruthenium(II) polypyridyl complexes caused much more damage in HCC1937 than in the MCF-7 and MDA-MB-231 cells, respectively. It was also notable that both ruthenium complexes blocked 50% DNA amplification of the BRCA1 exon 11 of HCC1937 cells at their IC_50_ concentrations (Figure 8C, Table 1) calculated by the RTCA assay. The MCF-7 cells seemed to be slightly more damaged than MDA-MB-231 cells up to 500 μM (Figure 8A).

### mRNA expression induced by the ruthenium(II) complexes

**Figure 9:**
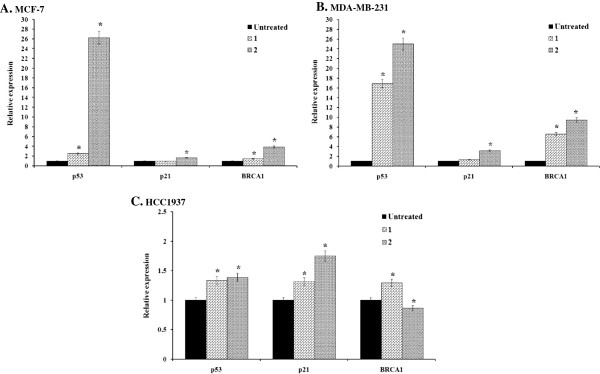
**mRNA determination.** MCF-7 **(A)**, MDA-MB-231 **(B)**, and HCC1937 **(C)** cells were treated with appropriate IC_50_ concentrations of **1** or **2** (Table 1) for 24 h. The transcript abundance of the genes was assessed by a real-time quantitative RT-PCR, normalized with the expression of β-Actin and relative to the expression of untreated control cells. Data were analyzed according to the 2^-∆∆C^_T_ method [27]. All experiments were performed in triplicate.

The real-time quantitative RT-PCR results of mRNA expression induced by **1** and **2** are shown in Figure 9A and B. The data indicated that **1** upregulated the expression of p53 and BRCA1 mRNA in all three cell lines but the expression of p21 mRNA in the MCF-7 and MDA-MB-231 cells was unchanged (*p* < 0.01). **2** upregulated the expression of p21 and p53 only in the MDA-MB-231 and MCF-7 cells but not BRCA1 mRNA in the BRCA1-defective HCC1937 cells (Figure 9C). It could be interpreted that **2** was capable of inhibiting the expression of BRCA1 mRNA. Of more interest was that the MDA-MB-231 and MCF-7 cells treated with **2** showed a significant 17-fold increase in the expression of p53 mRNA compared to the HCC1937 cells. The induction of p53 mRNA expression could be correlated with the apoptotic cell death. In this situation, it is likely that these cell lines use p53-dependent apoptosis pathways [4].

### Changes in secondary structure of the *N*-terminal BRCA1 RING protein induced by metallo-intercalator ruthenium(II) complexes

Presently, two main controversial aspects are whether ruthenium compounds target DNA or proteins. However, a major limitation in this framework is their unknown mode of action, and the lack of a specific biological target. Extensive investigations of the ruthenium-based compounds has mainly focused on the characterization of ruthenium-DNA adducts and has paid less attention to other potential cellular targets. For ruthenium-based drug candidates, their precise mechanisms of action as anticancer agents remain comparatively unexplored. There is evidence to indicate that ruthenium compounds might directly interfere with specific proteins involved in the signal transduction pathways, cell adhesion and migration processes [21,22]. However, the molecular mechanism and the signaling pathways still remain unidentified. Recently, a novel approach for cancer therapy involved alterations to the double strand breaks (DSBs) repair processes by which the cancerous cells with dysfunctional DNA repair pathways to accumulate high levels of DNA damage that eventually resulted in major genomic instability and cell death [31-35]. The breast cancer suppressor protein 1 (BRCA1)-mediated the DNA repair pathway, one of the important DNA damage response pathways, that plays a vital role in the maintenance of the genome integrity. Several lines of evidence have demonstrated that cancerous cells with inactivated BRCA1 had a defect in the repair of DSBs [36,37], and this conferred hypersensitivity to the cancerous cells towards platinum-based chemotherapy drugs and γ-irridation [24,25,38,39]. To our knowledge, the effect of the ruthenium(II) complexes on a DNA repair protein BRCA1 has not been studied. Therefore, it was of great interest to investigate the effect of the ruthenium(II) complexes with the bidentate ligand 5-chloro-2-(phenylazo)pyridine, **1** and **2**, on BRCA1 binding and its secondary structure.

**Figure 10:**
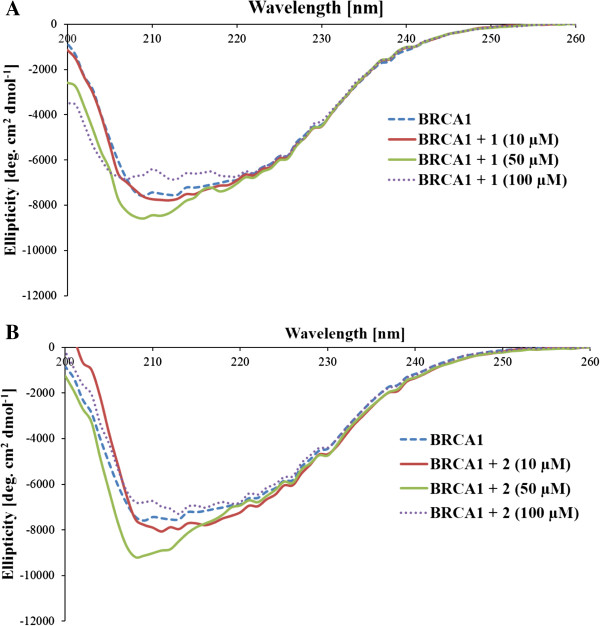
**CD spectra of ruthenium-BRCA1 adducts.** Ten μM of BRCA1 protein was pre-incubated with 30 μM of ZnCl_2_ at 4°C for 8 h. Samples were then incubated with the ruthenium(II) complexes in the dark at ambient temperature for 16 h before CD measurement at 25°C with a scanning rate of 50 nm/min. The mean residues ellipticity and wavelength that ranged from 200 to 260 nm were plotted. The CD spectra of the **1**-BRCA1 adducts **(A)**, **2**-BRCA1 adducts **(B)** at a number of concentrations that induced secondary structure changes of the holo-form of the BRCA1 protein.

**Figure 11:**
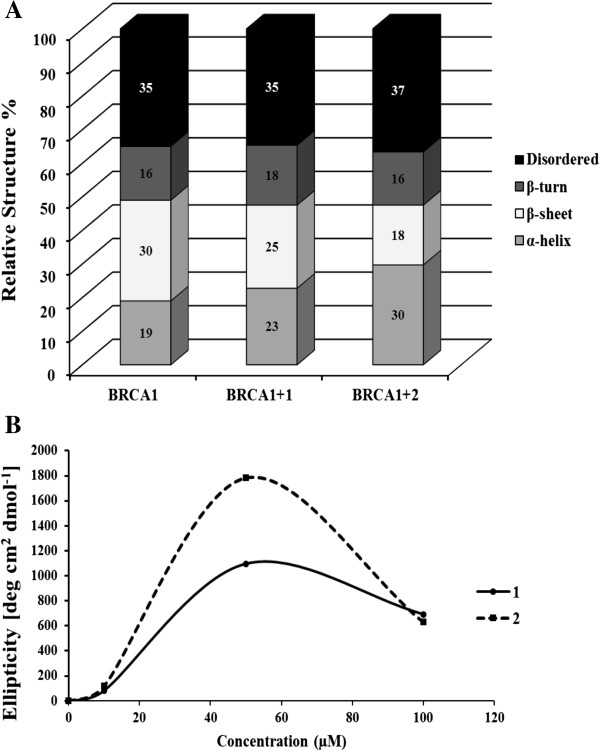
**The secondary structure and titration curve of the binding of metal complexes to the apo-form of the BRCA1 protein (without Zn**^**2+**^**). (A)** The effect of the ruthenium(II) complexes on the secondary structures of proteins was predicted by the CONTIN program. The relative secondary structure of the Ru-BRCA1 adducts at 50 μM of the complexes were plotted. **(B)** Changes in the ellipticity of the protein at 208 nm with increasing metal concentrations were plotted.

Circular dichroism (CD) was used to monitor the conformational changes of the *N*-terminal BRCA1 RING protein. The CD spectra of the Ru-induced holo-form of the BRCA1 RING protein (with bound Zn^2+^) showed similar profiles in shape with some differences in their amplitudes, and both proteins were maintained and underwent more folded structural rearrangements after the metal complex concentrations were increased, as judged by an increase in the negative CD spectra at 208 and 220 nm. In the present study, a **2**-induced BRCA1 RING showed a more enhanced increase in the negative CD spectra at 208 and 220 nm than for the **1**-induced BRCA1 RING (Figure 10A and B). This indicated that **2** can bind better to BRCA1 than **1**. Surprisingly, the CD spectra amplitudes of the BRCA1 RING proteins were obviously decreased in their negative CD spectra at 208 and 220 nm at high concentrations of both complexes (100 μM) when compared with the metal-free BRCA1 protein, and this indicated that both complexes interfered with the structural folding of the BRCA1 protein and induced protein aggregation. Based on the CONTIN program, the secondary structure of BRCA1 RING proteins were predicted (Figure 11A and B). Both complexes showed a similar increase in α-helical content and a decrease of β-sheets of the BRCA1 RING proteins. This indicated that the binding of metal complexes to proteins perturbed the secondary structure of the BRCA1 protein. In addition, the CD spectra amplitudes of the BRCA1 RING proteins were obviously decreased in their negative CD spectra at 208 and 220 nm at high concentrations of both complexes (100 μM) when compared with the control untreated proteins. This implied that the chelating interactions were disrupted, to cause a decrease in the CD spectra amplitudes of the protein [40,41]. In addition, the binding constant of the Ru-induced BRCA1 protein was found to be 6.53 ± 0.09 ×10^5^ M^-1^ with a free energy of binding (ΔG) of -252.95 cal mol^-1^ of the **2**-induced and 3.18 ± 0.05 × 10^5^ M^-1^ with the free energy of binding of 679.22 cal mol^-1^ of the **1**-induced, respectively.

### The *N*-terminal BRCA1 protein-mediated E3 ligase is inactivated by the ruthenium-based drugs

**Figure 12:**
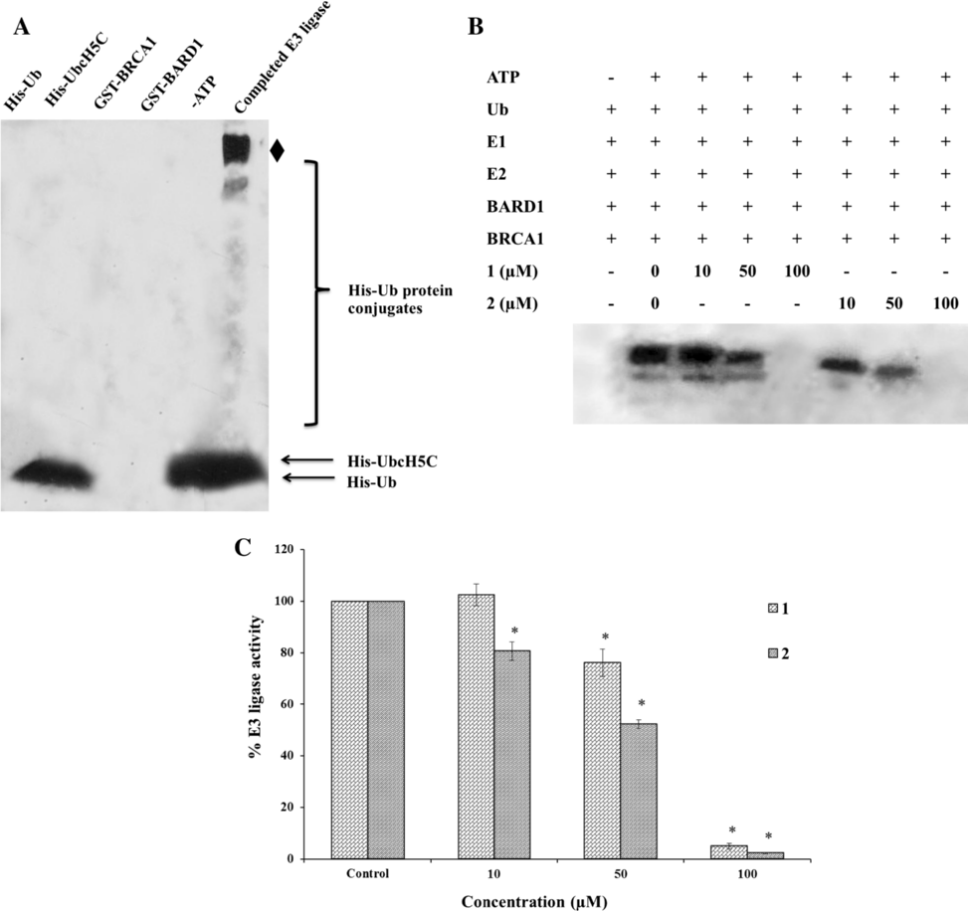
**In vitro ubiquitination.** The reactions, lacking a defined reactant, were incubated at 37°C for 3 h, and assayed for ubiquitin ligase activities. The reactions were terminated by adding an equal volume of SDS-loading dye before electrophoresis on 10% SDS/PAGE. The separated protein was then transferred to a PVDF membrane and immunodetected with conjugated anti-His_**6**_ Horseradish Peroxidase. The blot was detected by chemiluminescence on an X-ray film (see materials and methods). The loading control of E3 ligase activity and an apparent ubiquitination product was indicated by filled diamond **(A)**. The effects of **1** and **2** on E3 ligase activity were compared with the non-treated control BRCA1 **(B)**. The relative E3 ligase activity of the BRCA1 adducts was quantified by normalizing the density of an apparent band of the ubiquitinated-protein conjugates to that of the parental BRCA1 as the control, using a Bio-Rad GS-700 Imaging Densitometer. The relative E3 ligase activity of the BRCA1 adducts (%) was plotted as a function of the concentration of the ruthenium(II) polypyridyl complexes **(C)**. The significant reduction of E3 ligase activity of the BRCA1 adducts induced by **1** or **2** was compared using one-way ANOVA (^*^ = *p* < 0.01), relative to control.

To achieve a potentially targeted therapy with this class of ruthenium-based drugs, the BRCA1 RING domain protein has been used for an *in vitro* functional assay of BRCA1-mediated E3 ubiquitin ligase activity induced by **1** and **2**. The E3 ligase activity decreased as the concentration of ruthenium complexes increased. Both **1** and **2** are promising agents that can interfere with the E3 ligase activity (Figure 12B and C). However, **2** exhibited a slightly higher ability to inhibit E3 ligase activity than **1**. The E3 ligase activity was reduced by half at concentrations of 50 μM for 2, and 70 μM for 1, respectively.

## Discussion

Recently, EGFR, mTOR, and Poly (ADP-ribose) polymerase (PARP) inhibitors were among the therapeutic agents being studied in patients with TNBC and BRCA1-associated breast cancers [4]. However, the outcomes of those inhibitors have not been successful. Therefore, the development of new targeted therapies for TNBC is clearly needed to help this patient population. It has been reported that different platinum-based regimens showed higher chemosensitivity against TNBC compared with other breast cancer subtypes, both in the neoadjuvant and metastatic settings [8,42]. In addition, treatment with platinum-based drugs alone or in combination with other anticancer compound has improved outcomes for TNBC patients. However, the platinum drugs are limited by their severe toxicity and tendency to induce drug resistance [43]. Furthermore, cisplatin-induced secondary mutations in the tumors of BRCA1 mutation carriers have been shown to confer resistance to such a platinum-based drug [11,44]. For these reason, ruthenium-based drugs are being developed and have offered potential advantages over the antitumor platinum(II) complexes including reduced toxicity, a novel mechanism of action, the prospect of no cross-resistance and a different spectrum of activity [12]. Several lines of evidence have indicated that ruthenium polypyridyl complexes inhibit the proliferation of cells by inhibiting cell proliferation, cell cycle progression and inducing apoptosis [16,19,45,46].

In this study, we used triple-negative MDA-MB-231, BRCA1-mutated HCC1937 and sporadic BRCA1-competent MCF-7 cell lines as models of breast cancer cell growth and progression. Dynamic evaluation by the RTCA system showed that **1** and **2** quickly inhibited the proliferation of MCF-7, MDA-MB-231 and HCC1937 cancer cells within a few hours after treatment with the ruthenium complexes. This indicated a direct cytotoxic response towards these complexes. A continuous drop in the CI was observed at high concentrations of the complexes. Comparing the IC_50_ values of **1** and **2**, **2** appeared to have a higher cytotoxicity against all three breast cancer cell lines than **1**. Each ruthenium complex was differently absorbed by these cells and had distinct modulations on the arrest of cell cycle progression. **2** induced a significant block at the G2/M cell cycle arrest with a pronounced increase in apoptotic cells in the triple-negative MDA-MB-231 and BRCA1-defective cells. Our data may be attributed to the larger size, lipophilic characteristics of the polypyridyl ligand, and thereby enhancing their passage through the cell membrane. It has been reported that the intracellular uptake is to a major extent determined by the carrier ligand [15,18]. The hydrophobicity of the ruthenium-based drugs could also minimize the impact of the decreased accumulation of resistance mechanisms [19]. It has been shown that NAMI-A [47] inhibited the invasion and metastasis of cancer cells by arresting them at the G2/M stage and that it is a likely consequence of the accumulation of an inactive phosphorylated form of Cdk1, caused by the lack of Cdc25 phosphatase activity [21]. In addition, RAPTA-C inhibited cell proliferation by triggering the G2/M phase cell cycle arrest and the subsequent apoptosis [22]. It is well established that cell cycle progression is a tightly ordered and regulated process that involves multiple cellular checkpoints. These checkpoints respond to a variety of growth and transduction signals of the cells. In response to DNA damage, the checkpoints delay or stop in the cell cycle, at critical points before or during DNA replication (G1/S and intra S) and before cell division (G2/M).

A significant increase in apoptotic cells in the triple-negative MDA-MB-231 cells could result from an alternative breast cancer progression pathway defined through over-expression of the epidermal growth factor (EGF)-induced nuclear factor κB (NF-κB) that can be activated for ER- negative breast cancer cells [48]. NF-κB controls the cell-cycle progression by modulating the action of cell-cycle regulatory proteins. In addition, this triple-negative cell could trigger multiple pathways towards apoptosis, including those involving endonuclease G, caspases, and c-Jun *N*-terminal kinase [49]. MDA-MB-231 cells that lacked ER appeared to be less sensitive to tested metal-based drugs compared with its counterpart ER-harboring the MCF-7 cells. This data could be interpreted to mean that the ER plays a vital role in cell proliferation and cell viability of breast cancers. In the ER-positive breast cancer cells, ER exists in an inactive status as a complex with an inhibitory heat shock protein 90 (hsp90). Upon estrogen binding, hsp90 is released, and ER converts to an active conformation that interacts with its responsive DNA element, ERE. This ER-ERE interaction leads to the expression of hormone-responsive genes [50]. It was also a surprise, to find that HCC1937 cells that harbored a BRCA1 mutation (5382insC) and lacked an estrogen receptor were 10–20 fold more sensitive than the MDA-MB-231 cells. This implied that the increase in the ruthenium sensitivity in BRCA1 defective breast cancer cells might be related to a dysfunctional BRCA1 that is unable to repair DNA damage produced by ruthenium treatment, and ultimately led to breast cancer cell death [51]. Recently, the loss of BRCA1 led to an increase in the expression of the epidermal growth factor receptor (EGFR) in mammary epithelial cells, and inhibition of EGFR prevented ER-negative cancers in BRCA1-mutant mice [52].

In cancer cells, damage to the BRCA1 gene by the ruthenium-based drugs could lead to a loss of its functions and ultimately result in the death of the cancer cell. To address this concern, we further investigated whether these metallo-intercalator ruthenium complexes had any effect on cellular DNA damage that was affected by the BRCA1 gene. It was a surprise when both ruthenium complexes at their IC_50_ values caused a dramatic decrease in BRCA1 replication in HCC1937 cells while they produced only slight damage at equimolar concentrations or even at their IC_50_ concentration in MCF-7 and MDA-MB-231 cells. This may be in part due to the biology and cellular responses of each cancer subtype to ruthenium treatment [2,53]. This has provided the first evidence with regard to the inhibition of DNA replication of the BRCA1 gene in BRCA1-defective HCC1937 cells that was induced by this class of the ruthenium complexes, and this closely correlated to our previous result performed with the platinum-based drugs [54]. However, a lower dose of ruthenium complexes is required than for either cisplatin or carboplatin to achieve the 50% inhibition of DNA replication in such a cell line. A higher degree of inhibition of BRCA1 polymerization was induced by **2** in HCC1937 than in MCF-7 and MDA-MB-231 and this is most probably linked to the increased accumulation of **2** in the nuclear compartment of cancerous cells. Consequently, it can more easily damage the DNA and signal transduction pathways involved in cell cycle progression and apoptosis.

The quantitative RT-PCR data demonstrated that treatment with **1,** upregulated the expression of p53 and BRCA1 mRNA in all tested cell lines while **2** slightly downregulated the expression of BRCA1 mRNA in BRCA1-mutated HCC1937 cells. An increase in p53 mRNA reflected cellular responses to DNA-damaging agents, presumably to allow the cells to perform critical repair functions prior to passing through the cell cycle [55]. In this situation, it is likely that these cell lines use p53-dependent apoptosis pathways [4]. In the present study, it revealed that **2** did suppress or damage the BRCA1 gene in the HCC1937 cell lines, and the inhibition of transcription by **2** was a critical determinant of cell-cycle arrest in the G2/M phase because cells could not synthesize the mRNA necessary to pass into mitosis, and this eventually led to apoptosis. Low BRCA1 mRNA expression was correlated with an increased response rate and median overall survival in cisplatin-based chemotherapy or chemoradiotherapy [56]. This might lead to an insufficiency of BRCA1 function in cancer cells. In contrast, the up-regulation of BRCA1 mRNA induced by **1** in both MCF-7 and MDA-MB-231 cells could partly contribute to cellular resistance to **1**. In addition, the over-expression of BRCA1 mRNA in MCF-7 cells has also been reported to have increased resistance to cisplatin [57]. The levels of BRCA1 mRNA expression predicted outcomes following cisplatin-chemotherapy [58]. Cancer patients treated with cisplatin, or those with low or intermediate levels of BRCA1 mRNA also attained a significantly better response, disease-free survival and overall survival than those with high levels [59,60].

To further characterize whether these ruthenium complexes affected the BRCA1 protein, we performed ruthenium-BRCA1 interactions in vitro, and utilized the *N*-terminal BRCA1 RING domain protein (1–304 amino acid residues without mutating BRCA1 that play a vital role in E3 ubiquitin ligase activity) as a representative of all breast cancer cell lines used in this study. Our study on the Ru-BRCA1 interactions indicated that **1** and **2** both interacted with the holo-form of the BRCA1 protein and affected the overall conformation of the Zn^2+^-bound BRCA1. In addition, the effect of **2** was more effective than **1**. This might be attributable to the chemical structure of the phenanthroline ligand of **2** that is more planar and hydrophobic than the bipyridyl ligand of **1**. However, this observation is not the explanation for the anticancer platinum drug cisplatin [29]. In that case the affinity of Zn^2+^ or cisplatin to the holo-form of the BRCA1 RING protein has been demonstrated to occur in a different way. The Pt binding to BRCA1 had a binding constant of 3.00 ×10^6^ M^-1^, equivalent to that of the Zn^2+^ binding (2.79 ×10^6^ M^-1^). The calculated free energy of cisplatin and Zn^2+^ bindings were -8.68 and -8.64 kcal mol^-1^, respectively. The difference of the binding constant and free energy can be explained by the compact structure of the protein molecule. In addition, changes in the structural consequences of some protein conformations in the presence of ruthenium complexes, such as the binding of the ruthenium(III) complex KP1019 to cytochrome *c* induced conformational changes in the protein. This conformational change was subsequently expected to play a vital role in the biological activity of cytochrome *c*, in particular, in its ability to induce cell apoptosis [23].

For BRCA1-mediated ubiquitination, a reduction of the BRCA1 E3 ligase activity by both ruthenium complexes could reflect an altered interaction between the RING heterodimer domains of BRCA1 and BARD1. A slight difference in the reactivity toward the BRCA1 RING domain protein may be due to the geometry of each ruthenium complex [15]. This observation also agreed very well with the above mentioned ruthenium binding to the *N*-terminal BRCA1 RING domain protein. It is also notable that inactivation of BRCA1 E3 ligase activity induced by **1** and **2** was similar to that induced by the platinum-based drugs [24]. BRCA1 plays a vital role in the maintenance of genomic integrity through multiple functions including DNA damage repair, transcriptional regulation, a cell cycle checkpoint and protein ubiquitination. Cancerous cells with inactivated BRCA1 had defects in the repair of DNA double strand breaks (DSBs). These cells have increased sensitivity to DNA-damaging agents that eventually result in major genomic instability and cell death. Several investigations have revealed the relevance of the BRCA1-mediated ubiquitination to DNA repair functions. Mutations in the BRCA1 RING domain resulted in a loss of the E3 ubiquitin ligase activity, and conferred hypersensitivity of the cancerous cells to chemotherapy that causes DNA damage [24,25,61]. Therefore, targeting the BRCA1 RING domain protein through the disruption of the BRCA1 E3 ligase activity by this class of ruthenium complexes might be an effective approach to eradicate breast cancers. However, future trials need to be considered before utilizing BRCA1 as a promising therapeutic target for breast cancer treatment.

## Conclusions

This study revealed the ability of ruthenium complexes to inhibit cell proliferation, induce cell cycle progression and apoptosis. Ruthenium treatment upregulated the marker genes involved in apoptosis and cell cycle progression while it downregulated BRCA1 mRNA and replication of BRCA1-defective breast cancer cells. Changes in conformation and the binding constant of ruthenium-BRCA1 adducts were observed, causing inactivation of the RING heterodimer BRCA1/BARD1-mediated E3 ubiquitin ligase activity. Our results could provide an alternative approach to finding effective therapeutic ruthenium-based agents with promising anticancer activity, and have identified the BRCA1 RING domain protein as a promising therapeutic target for breast cancers.

## Abbreviations

BARD1: BRCA1-associated RING domain 1; BLBC: Basal-like breast cancer; BRCA1: Breast cancer susceptibility gene 1; BSA: Bovine serum albumin; CD: Circular dichroism; DSBs: DNA double strand breaks; DMEM: Dulbecco’s modified Eagle’s medium with 10% fetal bovine serum; EGFR: Epidermal growth factor receptor; ER: Estrogen receptor; ERE: Estrogen responsive DNA element; FBS: Fetal bovine serum; HER2: Human epidermal growth factor receptor 2; HR: Homologous recombination repair; hsp: Heat shock protein; ICP-MS: Inductively coupled plasma-mass spectrometer; mTOR: Mammalian target of rapamycin; PARP: Poly (ADP-ribose) polymerase; PBS: Phosphate-buffered saline; pCR: Pathologic complete response; PI: Propidium iodide; PR: Progesterone receptor; QPCR: Quantitative polymerase chain reaction; RPMI 1640: Roswell Park Memorial Insititute 1640 medium; RTCA: Real-time cellular analyzer; RT-PCR: Reverse transcription polymerase chain reaction; Ru: Ruthenium; SDS-PAGE: Sodium dodecyl sulphate-polyacrylamide gel electrophoresis; TNBC: Triple-negative breast cancer.

## Competing interests

The authors declare that they have no competing interests.

## Authors’ contributions

TN conducted cellular uptake, cell cycle arrest, apoptosis, QPCR, real-time RT-PCR as well as data analysis. PT conducted cell proliferation, CD, in vitro ubiquitination, as well as real-time cellular analysis. KH generated the ruthenium ruthenium(II) complexes with the Clazpy ligand, [Ru(Clazpy)_2_bpy]Cl_2_.7H_2_O (**1**) and [Ru(Clazpy)_2_phen]Cl_2_.8H_2_O (**2**) and the editing of the manuscript. AR conceived the study, participated in the concept and design of the study and in revising and editing the manuscript. All authors have read and approved the final manuscript.

## Pre-publication history

The pre-publication history for this paper can be accessed here:

http://www.biomedcentral.com/1471-2407/14/73/prepub

## Supplementary Material

Additional file 1: Table S1 Supplementary Information. Click here for file
