# Supplementary material for: Cellular responses of BRCA1-defective and triple-negative breast cancer cells and in vitro BRCA1 interactions induced by metallo-intercalator ruthenium(II) complexes containing chloro-substituted phenylazopyridine
Source: BMC Cancer. 2014 Feb 7;14:73. doi: 10.1186/1471-2407-14-73 (PMC3933379; doi:10.1186/1471-2407-14-73)
Supplement: Additional file 1: Table S1 — Supplementary Information. [file 1471-2407-14-73-S1.doc]

**Supplementary Information (SI)**

**Table S1** IC_50_ values (µM) of MCF-7, MDA-MB-231 and HCC1937 cells after **1**, **2**, and cisplatin treatment. Cells were treated with increasing concentrations of either **1** or **2**, and cisplatin and an MTT assay was performed after 24 h (data reflect the mean and ±SD of results from three separate experiments, each performed in triplicate). Statistical significance differences are indicated by **p*<0.01, compared the IC_50_ values of the same complex on cell lines, and ***p*<0.01, compared the IC_50_ values of the complexes on each cell line.

| Metal complexes | **IC_50_ (µM)** | | |
| --- | --- | --- | --- |
|  | MCF-7 | MDA-MB-231 | HCC1937 |
| Cisplatin ^a^ | 53.9 ± 0.5 ^*,**^ | > 150 | 22.9 ± 0.3 ^*,**^ |
| **1** | 18.4 ± 0.1 ^*,**^ | 39.2 ± 0.2 ^*,**^ | 6.1 ± 0.2 ^*,**^ |
| **2** | 15.1 ± 0.3 ^*,**^ | 36.1 ± 0.3 ^*,**^ | 4.6 ± 0.1 ^*,**^ |

^a^ IC_50_ value of cisplatin after 48 h of treatment.
